# Strongyloidiasis and Culture-Negative Suppurative Meningitis, Japan, 1993–2015

**DOI:** 10.3201/eid2412.180375

**Published:** 2018-12

**Authors:** Mitsuru Mukaigawara, Izumi Nakayama, Koichiro Gibo

**Affiliations:** Okinawa Chubu Hospital, Okinawa, Japan

**Keywords:** Strongyloidiasis, meningitis/encephalitis, HTLV-1, culture-negative suppurative meningitis, Enterobacteriaceae, Okinawa, Japan, parasites, bacteria

## Abstract

Community-acquired *Enterobacteriaceae* infection and culture-negative meningitis are rare and atypical subtypes of meningitis in adults. Of 37 patients who had atypical suppurative meningitis during 1993–2015 in Okinawa, Japan, 54.5% had strongyloidiasis, of which 9.1% cases were hyperinfections and 3.0% dissemination. Strongyloidiasis should be considered an underlying cause of atypical suppurative meningitis.

Among adults, suppurative meningitis caused by enteric organisms and suppurative meningitis that is culture negative are uncommon (*1*,*2*). These types of meningitis with atypical features (hereafter atypical suppurative meningitis) remain a clinical challenge. The mortality rate among patients with community-acquired suppurative meningitis caused by gram-negative organisms is 52.5% (*1*). Treatment of culture-negative suppurative meningitis requires broad-spectrum antimicrobial drugs; however, the absence of detected pathogens increases the risk for development of antimicrobial drug resistance.

Strongyloidiasis, a nematode infection that occurs in the subtropics and tropics, is associated with *Enterobacteriaceae* meningitis (*3*). Previous reports of strongyloidiasis-associated meningitis also suggested potential links between strongyloidiasis and atypical suppurative meningitis on the basis of 9 cases of *Enterobacteriaceae* meningitis (not consecutive) (*4*) and 17 cases of culture-negative suppurative meningitis (*5*). Our aim was to investigate the association between strongyloidiasis and atypical suppurative meningitis.

We conducted a retrospective chart review of patients who consecutively received a diagnosis of atypical suppurative meningitis during January 1993–December 2015 at Okinawa Chubu Hospital, Okinawa, Japan. This hospital is one of the largest tertiary medical centers in Okinawa. Strongyloidiasis is endemic in Okinawa; reported prevalence is 5.2% (*6*).

We defined atypical suppurative meningitis as suppurative meningitis with positive cerebrospinal fluid (CSF) culture results for enteric organisms or with CSF leukocytosis >500 cells/mm^3^ and negative CSF culture results. We included in the study patients >18 years of age with CSF that was culture positive for enteric organisms or negative with leukocytosis of >500 cells/mm^3^. Enteric organisms included in this study were *Bacteroides* spp., *Enterococcus* spp., *Escherichia coli*, *Enterobacter* spp., *Klebsiella* spp., *Bifidobacterium bifidum*, *Clostridium perfringens*, *Proteus mirabilis*, *Streptococcus gallolyticus* (*bovis*), and *Campylobacter* spp. (*7*). CSF of patients with bacterial meningitis typically shows leukocytosis of >1,000 cells/mm^3^; CSF of those with nonbacterial meningitis typically shows <250 cells/mm^3^ (*8*). Considering the early phase of bacterial meningitis (*9*), the cutoff value (500 cells/mm^3^) was defined to include suppurative meningitis and exclude most cases of nonbacterial meningitis. We excluded patients with nosocomial meningitis, prior use of antimicrobial drugs (within 7 days of lumbar puncture), negative CSF culture, and positive blood culture for nonenteric organisms.

We collected information about patient demographic and clinical characteristics, immunocompromised status, type of strongyloidiasis infection, outcomes, CSF analysis results, and culture results. Strongyloidiasis was classified into 3 categories: nonsystemic strongyloidiasis, hyperinfection, and dissemination. We defined these categories according to where larvae were detected: nonsystemic strongyloidiasis in fecal samples only, hyperinfection in sputum, and dissemination in samples other than feces or sputum (*4*). Samples were collected with regard to patients’ clinical category. Identifying information was removed before analysis. This study was approved by the Okinawa Chubu Hospital Institutional Review Board (H29–76).

We identified 37 patients; CSF culture results were positive for *Enterobacteriaceae* for 14 and negative for 23. Strongyloidiasis was diagnosed by parasitologic examinations (Table; Technical Appendix).

**Table:** Demographic, clinical, and laboratory characteristics of patients with atypical suppurative meningitis, Japan, 1993–2015*

Characteristic	All cases, no. (%), n = 37	Culture-positive, no. (%), n = 14	Culture-negative, no. (%), n = 23
Demographic and clinical			
Sex			
M	22 (59.5)	9 (64.2)	13 (56.5)
F	15 (40.5)	5 (35.8)	10 (43.5)
Chief complaints			
Headache	24 (64.9)	6 (42.9)	18 (78.3)
Fever	22 (59.5)	9 (64.2)	13 (56.5)
Altered mental status	8 (21.6)	5 (35.7)	3 (13.0)
Nausea/vomiting	8 (21.6)	1 (7.1)	7 (30.4)
Immunocompromised status			
HTLV-1 infection†	22 (71.0)	8 (61.5)	14 (77.8)
Diabetes mellitus	3 (8.1)	2 (14.3)	1 (4.3)
Cirrhosis	3 (8.1)	3 (21.4)	0
Steroid use	4 (10.8)	1 (7.1)	3 (13.0)
Strongyloidiasis infection type			
Nonsystemic strongyloidiasis	14 (42.4)	5 (35.7)	9 (47.4)
Hyperinfection	3 (9.1)	3 (21.4)	0
Dissemination	1 (3.0)	0	1 (5.3)
Not analyzed	4	0	4
Died	5 (13.5)	3 (21.4)	2 (8.7)
CSF			
Neutrophils/mm^3^			
500–2,999	24 (64.9)	6 (42.9)	18 (78.3)
3,000–5,999	4 (10.8)	1 (7.1)	3 (13.0)
6,000–9,999	2 (5.4)	1 (7.1)	1 (4.3)
>10,000	3 (8.1)	2 (14.3)	1 (4.3)
Glucose <40 mg/dL	14 (37.8)	7 (50.0)	7 (30.4)
Bacteriologic			
Blood culture			
* Klebsiella pneumoniae*	7 (18.9)	5 (35.7)	2 (8.7)
* Streptococcus gallolyticus*	4 (10.8)	4 (28.6)	0
* Escherichia coli*	3 (8.1)	2 (14.3)	1 (4.3)
* Streptococcus infantarius*	1 (2.7)	1 (7.1)	0
* Campylobacter fetus*	1 (2.7)	1 (7.1)	0
Negative	21 (56.8)	1 (7.1)	20 (87.0)
CSF culture			
* K. pneumoniae*	5 (35.7)	5 (35.7)	0
* S. gallolyticus*	1 (7.1)	1 (7.1)	0
* E. coli*	1 (7.1)	1 (7.1)	0
* S. infantarius*	1 (7.1)	1 (7.1)	0
* C. fetus*	1 (7.1)	1 (7.1)	0
* Bacteroides fragilis*	1 (7.1)	1 (7.1)	0

*Patient median age (range): all, 60 y (19–91 y); culture-positive, 57 y (27–91 y); culture-negative, 58 y (19–90 y). CSF, cerebrospinal fluid; HTLV-1, human T-cell lymphotropic virus type 1.  †HTLV-1 serology results were available for 93% (13/14) of culture-positive patients and 78% (18/23) of culture-negative patients.

Patients were 19–91 years of age (median 60 years of age); 59.5% (22/37) were male. Common chief complaints included headache (64.9% [24/37]) and fever (59.5% [22/37]). Human T-cell lymphotropic virus type 1 serologic test results were available for 31 patients, of which 22 (71.0%) were positive. Of the 37 total patients, 3 (8.1%) patients had diabetes mellitus, 3 (8.1%) had cirrhosis, and 4 (10.8%) used steroids on a regular basis. Parasitologic examinations were performed for 33 patients, and strongyloides were found in 18 (54.5%): 14 (42.4%) nonsystemic strongyloidiasis, 3 (9.1%) hyperinfection, and 1 (3.0%) dissemination. 

Among 14 culture-positive patients, 5 (35.7%) had nonsystemic strongyloidiasis and 3 (21.4%) had hyperinfection. Among 19 culture-negative patients, 9 (47.4%) had nonsystemic strongyloidiasis and 1 (5.3%) had dissemination. When culture-positive patients with strongyloidiasis were compared with culture-negative patients with strongyloidiasis, the odds ratio was 1.71 (95% CI 0.37–8.22). Of note, all strongyloidiasis-positive patients were born before 1960, suggesting changes in lifestyle and the environment since then (e.g., reduced exposure to contaminated soil during farming by not walking barefoot and improved farming environments).

In patients with culture-positive meningitis, blood and CSF culture results were positive for *Klebsiella pneumoniae*, *S. gallolyticus*, *E. coli*, *Streptococcus infantarius*, and *Campylobacter fetus*. Among patients with culture-negative meningitis, blood culture results were positive for *K. pneumoniae* and *E. coli*.

Our investigation has several limitations because of the single-center, retrospective nature of this study. Also, the sensitivity of parasitologic examination is low (*10*). We potentially underestimated the prevalence of strongyloidiasis.

On the basis of previous reports of associations between strongyloidiasis and *Enterobacteriaceae* meningitis (*3**,**5*), our analysis proposes an association between strongyloidiasis and culture-negative suppurative meningitis in Okinawa. Our findings suggest that atypical suppurative meningitis can occur as occult dissemination (*3**,**4*), that anthelminthic treatment may be indicated, and that steroids should be administered with caution (*5*). The presence of atypical suppurative meningitis in adults should prompt consideration of occult disseminated strongyloidiasis; the index of suspicion for patients with atypical suppurative meningitis is high.

Technical Appendix Additional methods and results for study of strongyloidiasis and culture-negative suppurative meningitis, Okinawa, Japan, 1993–2015.
