# Supplementary material for: Strongyloidiasis and Culture-Negative Suppurative Meningitis, Japan, 1993–2015
Source: Emerg Infect Dis. 2018 Dec;24(12):2378–80. doi: 10.3201/eid2412.180375 (PMC6256405; doi:10.3201/eid2412.180375)
Supplement: Technical Appendix — Additional methods and results for study of strongyloidiasis and culture-negative suppurative meningitis, Okinawa, Japan, 1993–2015. [file 18-0375-Techapp-s1.pdf]

Article DOI: https://doi.org/10.3201/eid2412.180375

Strongyloidiasis and Culture-Negative Suppurative Meningitis, Japan, 1993–2015

Technical Appendix

We extracted the data of patients with strongyloidiasis from our cohort, the results of which are shown in the appendix table. The age ranged from 40 to 83 years (median, 57.5 years). All patients were born in or before the year 1960. Among 18 patients with strongyloidiasis, 83.3% (15 patients) were male. Common chief complaints included headache, fever, altered mental status, and nausea and vomiting. Seventeen patients (94.4%) were found positive for HTLV-1 infection. Of 18 patients with strongyloidiasis, 8 (44.4%) presented with culture-positive meningitis, and 10 (55.6%) culture-negative. Culture results were positive for *Klebsiella pneumoniae*, *Streptococcus gallolyticus*, and *Streptococcus infantarius*.

Technical Appendix Table. Demographic, clinical, and laboratory characteristics of patients with strongyloidiasis, Japan, 1993–2015*

Characteristic	Strongyloidiasis-positive patients (n = 18)
Demographic and clinical characteristics	
Age, median (range)	57.5 (40–83)
Birth year (%)	
1910–19	1 (5.6)
1920–29	3 (16.7)
1930–39	2 (11.1)
1940–49	4 (22.2)
1950–59	6 (33.3)
1960–69	2 (11.1)
1970–	0 (0)
Male (%)	15 (83.3)
Chief complaints (%)	
Headache	11 (61.1)
Fever	8 (44.4)
Altered mental status	4 (22.2)
Nausea/vomiting	4 (22.2)
Immunocompromised status (%)	
HTLV-1 infection	17 (94.4)
Diabetes mellitus	0 (0)
Cirrhosis	1 (5.6)
Steroid use	1 (5.6)
Mortality (%)	3 (16.7)
Type of associated meningitis (%)	
Culture-positive	8 (44.4)
Culture-negative	10 (55.6)
CSF characteristics	

Characteristic	Strongyloidiasis-positive patients (n = 18)
Neutrophil count (%)	
500–2,999/mm^3	10 (55.6)
3,000–5,999/mm^3	2 (11.1)
6,000–9,999/mm^3	2 (11.1)
$\geq$10,000/mm^3	2 (11.1)
Glucose <40mg/dL (%)	10 (55.6)
Bacteriological characteristics	
Blood culture (%)	
Klebsiella pneumoniae	5 (27.8)
Streptococcus gallolyticus	4 (22.2)
Streptococcus infantarius	1 (5.6)
Negative	9 (50.0)
CSF culture (%)	
K. pneumoniae	2 (11.1)
S. gallolyticus	5 (27.8)
S. infantarius	1 (5.6)
Negative	10 (55.6)

*CSF, cerebrospinal fluid HTLV-1; human T-cell lymphotropic virus type 1.
